# Abnormalities associated with progressive aortic vascular dysfunction in chronic kidney disease

**DOI:** 10.3389/fphys.2015.00150

**Published:** 2015-05-19

**Authors:** Omar Z. Ameer, Rochelle Boyd, Mark Butlin, Alberto P. Avolio, Jacqueline K. Phillips

**Affiliations:** Faculty of Medicine and Health Sciences, The Australian School of Advanced Medicine, Macquarie UniversitySydney, NSW, Australia

**Keywords:** aorta, vasoconstriction, vasodilation, endothelium, nitric oxide, chronic kidney disease

## Abstract

Increased stiffness of large arteries in chronic kidney disease (CKD) has significant clinical implications. This study investigates the temporal development of thoracic aortic dysfunction in a rodent model of CKD, the Lewis polycystic kidney (LPK) rat. Animals aged 12 and 18 weeks were studied alongside age-matched Lewis controls (total *n* = 94). LPK rodents had elevated systolic blood pressure, left ventricular hypertrophy and progressively higher plasma creatinine and urea. Relative to Lewis controls, LPK exhibited reduced maximum aortic vasoconstriction (R_max_) to noradrenaline at 12 and 18 weeks, and to K^+^ (12 weeks). Sensitivity to noradrenaline was greater in 18-week-old LPK vs. age matched Lewis (effective concentration 50%: 24 × 10^−9^ ± 78 × 10^−10^ vs. 19 × 10^−8^ ± 49 × 10^−9^, *P* < 0.05). Endothelium-dependent (acetylcholine) and -independent (sodium nitroprusside) relaxation was diminished in LPK, declining with age (12 vs. 18 weeks R_max_: 80 ± 8% vs. 57 ± 9% and 92 ± 6% vs. 70 ± 9%, *P* < 0.05, respectively) in parallel with the decline in renal function. L-Arginine restored endothelial function in LPK, and L-NAME blunted acetylcholine relaxation in all groups. Impaired nitric oxide synthase (NOS) activity was recovered with L-Arginine plus L-NAME in 12, but not 18-week-old LPK. Aortic calcification was increased in LPK rats, as was collagen I/III, fibronectin and NADPH-oxidase subunit p47 (phox) mRNAs. Overall, our observations indicate that the vascular abnormalities associated with CKD are progressive in nature, being characterized by impaired vascular contraction and relaxation responses, concurrent with the development of endothelial dysfunction, which is likely driven by evolving deficits in NO signaling.

## Introduction

Individuals with chronic kidney disease (CKD) are at high risk of cardiovascular disease (Kuznik et al., 2013) with renal failure and the accumulation of uraemic toxins proposed to stimulate oxidative stress and inflammation that in turn may contribute to endothelial dysfunction (Schiffrin et al., 2007). Endothelial dysfunction in CKD manifests primarily as reduced endothelium-dependent vasodilation (Morris et al., 2000). Reduced nitric oxide (NO) signaling is believed to be one of the main factors involved in chronic renal failure–induced endothelial dysfunction (Hasdan et al., 2002) and may be caused by various mechanisms including decreased NO synthesis, increased NO degradation due to oxidative stress (Fuster et al., 1992; Quyyumi, 1998) or decreased NO-dependent soluble guanylate cyclase protein activation and cyclic guanosine monophosphate (cGMP) production (Giles et al., 2012).

Clinically significant changes in large artery viscoelasticity resulting in altered reactivity, reduced compliance and increased arterial stiffness have been reported among haemodialysis patients (London et al., 1990) and damage to the large capacitive arteries like the aorta is considered an important factor contributing to increased morbidity and mortality in end stage renal disease (ESRD) (Blacher et al., 1999; Pannier et al., 2005; Guérin et al., 2008). This suggests a pivotal role for the aorta in driving both systemic and/or organ dysfunction. Specifically, aortic wall changes affect vessel compliance and therefore pulse pressure. The transmission of this pulsatile energy is associated with end organ damage (O'Rourke and Safar, 2005). Increased pulse pressure, remodeling and stiffness of large arteries has been associated with the rate of change in renal function (Ford et al., 2010; Briet et al., 2011) including changes in glomerular filtration rate and microalbuminuria (Fesler et al., 2007; Hermans et al., 2007), and likely therefore contributes to the progression of CKD. Accordingly, aortic structure and function, as a crucial component of the cardiovascular system, can affect and be affected by kidney disease (Bakris et al., 2003; Mitchell, 2008).

The Lewis polycystic kidney (LPK) rat is an autosomal recessive model of cystic renal disease, arising from a mutation in the *nek8* gene (McCooke et al., 2012), and therefore a form of nephronophthisis (NPHP9), which in rodents has a phenotypic renal presentation resembling human autosomal recessive polycystic kidney disease (Phillips et al., 2007; Trapp et al., 2008). We have previously verified in the LPK rat aorta features of arteriosclerosis, including vascular remodeling, and calcification at 12 weeks of age, a time point where hypertension is established and rats manifest impaired renal function (Ng et al., 2011). Using tensile testing to assess the passive biomechanical properties of the aorta, and pulse wave velocity as a surrogate measure of arterial stiffness, we have evidence to indicate these structural changes result in functional abnormalities (Ng et al., 2011; Ameer et al., 2014), however no *in-vitro* assessment of large artery function has been made, nor has the relationship with ongoing deterioration in renal function been determined.

In the present investigation, we therefore aimed to determine if hypothesized changes in vascular contractility, endothelial-dependent and independent-mechanisms, and integrity of endothelial NO function in the LPK aorta were progressive in nature, examining animals at both intermediate and late time points in the disease process. We further examined other systems potentially underlying aortic vascular dysfunction including markers of stiffness, oxidative stress, calcification and systemic NO levels.

## Materials and methods

### Animals

Mixed sex 12 and 18 week old LPK and Lewis control rats were used in this study. Animals from three specific cohorts were used for the following analysis: (i) 12- and 18-week-old LPK and Lewis rats (*n* = 8 per age and strain) were used for *in-vitro* organ bath studies. Plasma analysis for urea, creatinine and plasma nitrates were determined for this cohort; (ii) 12- and 18-week-old LPK and Lewis rats (*n* = 4 per age and strain) were used for aortic mRNA analysis. Phenotypic data including bodyweight, systolic blood pressure (SBP), heart, left ventricle and kidney indices (tissue weight (g)/body weight (g) × 100%) and plasma analysis for urea and creatinine was determined for these animals. To facilitate tissue sharing and reduction in animal usage, some data from animals in this cohort (SBP, plasma urea and creatinine) contributed to another larger study correlating these variables with anaemia measures in the LPK model (Phillips et al., in press). (iii) 12- and 18-week-old LPK (*n* = 9 each age), and 12- and 18-week-old Lewis rats (*n* = 16 and 12, respectively) were used for aortic Ca^+2^ content analysis. A total of 94 rats were used overall. All animals were obtained from the Animal Resource Center (Perth, WA, Australia) and housed at Macquarie University (NSW, Australia). The rats were allowed to acclimatize in the animal house facility under standard 12/12 light-dark cycle (20.5°C) for at least 1 week prior to the experiments. The rats were fed with normal rat chow and water *ad libitum*. All experiments were approved by the Animal Ethics Committee of Macquarie University and carried out in accordance with the Australian Code of Practice for the Care and Use of Animals for Scientific Purposes (8th Edition, 2013).

### Tail-cuff plethysmography

Systolic blood pressure was measured using tail-cuff plethysmography (IITC Life Science Inc., CA, USA) 1–2 days prior to euthanasia, as previously described (Phillips et al., 2007). An average of 6 sequential measurements were taken after acclimatizing the animal to the restrainer.

### Animal euthanasia and plasma collection

Prior to euthanasia, animals were fasted overnight with free access to drinking water. Animals were then deeply anaesthetized with 5% isoflurane in 100% O_2_ and decapitated. Trunk blood samples were collected in pre-cooled EDTA-containing tubes and centrifuged (4°C at 3000 rpm for 5 min). Plasma was separated and stored at −80°C until further use.

### *In-vitro* aortic ring preparation

After euthanasia, the thoracic aorta was removed and placed in ice-cold Krebs solution (in mM: NaCl 118.2, KCl 4.7, CaCl_2_ 2.5, MgSO_4_ 1.2, KH_2_PO_4_ 1.2, glucose 11.7, NaHCO_3_ 25, and EDTA 0.026) continuously aerated with carbogen (5% CO_2_ + 95% O_2_). After clearing of adherent connective and adipose tissue, an aortic ring 3–5 mm in length was taken approximately 6 mm caudal to the aortic arch. The segment was horizontally mounted in an organ bath on 2 stainless steel hooks attached to a metal holder (Radnoti LLC, CA, USA) and maintained in Kreb's solution at 37.4°C, pH 7.3–7.5, constantly bubbled with carbogen. Rings were subjected to a 1 g (980 mN) tension and allowed to equilibrate for at least 40 min. During the equilibration period, Kreb's solution in the chamber was changed every 15 min to avoid metabolite build-up and tension was readjusted to 1 g if necessary as previously described (Ameer et al., 2010). Responses were recorded isometrically via a force displacement transducer connected to a PowerLab digital acquisition system and data was acquired using LabChart Software (both ADInstruments, CA, USA).

### Drugs

Noradrenaline hydrochloride (NA), phenylephrine hydrochloride (PE), acetylcholine hydrochloride (ACh), sodium nitroprusside (SNP), L-arginine (L-Arg), N^ω^ -nitro-L-arginine methyl ester hydrochloride (L-NAME) were purchased from Sigma-Aldrich (NSW, Australia). Drugs were made to a stock solution in 0.9% saline and then diluted to the required working concentration in Kreb's solution prior to addition to the organ bath or in the organ bath itself.

### *In-vitro* experimental protocols

The following experimental protocols were performed consecutively on one aortic ring from each rat, with at least a 20 min recovery period allowed between different drug conditions:

#### Protocol I

Vascular contractility was assessed by generating concentration-response curves to the α-adrenergic receptor agonist NA and depolarization induced by potassium chloride (KCl), reflecting aortic adrenergic and smooth muscle reactivity, respectively. Cumulative concentration-responses to final bath concentrations of 1 × 10^−10^–3 × 10^−4^M NA and 5–100 mM KCl were performed.

#### Protocol II

Endothelium-dependent and -independent relaxation responses were then assessed. Each ring was precontracted with 1 μM of the α_1_-adrenergic receptor agonist PE. Upon achieving the maximum plateau response, endothelium-dependent relaxation was assessed using cumulative concentrations (1 × 10^−10−1 × 10^−4^^M) of ACh. Tissues were then washed and following recovery (minimum 20 min), the rings were precontracted with PE and endothelium-independent relaxation evaluated using 1 × 10^−11^–1 × 10^−5^M cumulative concentrations of SNP.

#### Protocol III

The final experimental protocol was designed to assess endothelial function and the integrity of components of the NO pathway. In separate conditions, cumulative-responses to ACh were recorded in aortic rings precontracted with PE (1 μM), preincubated for 20 min with: (1) the nitric oxide synthase (NOS) substrate, L-Arg (10 mM) (Sato et al., 1996); (2) the non-selective NOS inhibitor, L-NAME (10 μM); (3) or a combination of both L-Arg and L-NAME.

Time control experiments, comparing NA maximum contractile response (R_max_) over the course of the experimental protocol (on average 340 min) were conducted to ensure viability of the aortic rings.

### Vascular responses data analysis

Contractile force responses to NA and KCl were normalized to the wet weight of the individual ring measured after completion of the experiment, as previously described (Kauser et al., 1998; Takahashi et al., 2003), to give a measure in N/g. Concentration-response curves were fitted to a sigmoidal curve: Y = Lower plateau + (R_max_- Lower plateau)/(1+10^(LogEC_50_−X^) where Y is the force response and X the drug concentration. The 50% effective concentration (EC_50_) was used to evaluate sensitivity to vasoactive substances. The largest response induced by the agonists (NA, KCl, ACh, and SNP) was considered the R_max_ and % responses were calculated relative to it. The area under the cumulative concentration-response curve (area under curve: AUC) was also determined for each respective agonist, representative of the total vascular reactivity (Vedernikov et al., 1997; Peinado et al., 1998). The differences between R_max_ response to ACh and that in the presence of L-NAME were considered as the NO-dependent component of the ACh-induced response (Paulis et al., 2008).

### Plasma assays

Plasma creatinine and urea levels were determined using an IDEXX VetTest® Chemistry Analyzer (IDEXX, NSW, Australia).

Plasma NO^−^_2_ and NO^−^_3_ were determined using DetectX® NO colorimetric detection kit (Arbor assays, MI, USA) following the manufacturer's instructions and as described previously (Fujiwara et al., 2000). Briefly, the plasma was diluted 1:4 with the assay buffer, and then passed through 10-kDa ultrafilters, mixed with 25 μ l cofactor and 25 μ l nitrate reductase. After the plasma mixture had been incubated at room temperature for 5 min to convert NO^−^_3_ to NO^−^_2_, total NO was measured at 540 nm absorbance (iMark microplate reader BioRad, Japan) by reaction with Greiss reagent (sulfanilamide and naphthalene-ethylenediamine dihydrochloride). Amounts of NO^−^_2_ in the plasma were estimated by a standard curve obtained from enzymatic conversion of NaNO_3_ to NO^−^_2_. Lastly, plasma NO^−^_3_ was obtained by subtraction of total NO from NO^−^_2_ concentration.

### Aortic calcium content

After euthanasia, the descending thoracic aorta was collected, cleaned with saline and dried at 40°C in an oven for 40 min. To measure aortic calcium content, tissue was then incubated in 1 M HCl for 72 h and calcium was determined colorimetrically using 100 μM o-cresolphthalein complexone in 270 mM aminomethylpropanol buffer (pH 10.0) with 5.2 mM 8-hydroxyquinolone added to complex magnesium as per the work of Sutliff et al. (2011). Absorbance was measured at 575 nm (U700 spectrophotometer, Beckman Coulter, CA, USA). Aortas were dried after extraction and weighed, and results were expressed as μmole per gram of dry weight (Sutliff et al., 2011).

### RNA isolation, reverse transcription, and quantitative polymerase chain reaction

After euthanasia, the descending thoracic aorta was dissected and total RNA was extracted using an RNA isolation kit (Master Pure RNA Purification Kit, Epicenter Biotechnologies, WI, USA), according to the manufacturer's protocol. RNA concentration was determined using a Nanodrop 2000 spectrophotometer (Thermo Fisher Scientific, VIC, Australia) and first-strand cDNA was synthesized from total RNA (2.5 μg, average 260/280 ratio 2 ± 0.1) using the Affinity Script TM QPCR cDNA Synthesis Kit (Stratagene, Agilent Technologies, CA, USA) using random primers (100 ng/μl), as per the manufacturer's instructions. Real time quantitative polymerase chain reaction (qPCR) was performed using 1 μl of cDNA mix in a 25 μl reaction with each forward and reverse primer (300–600 nM final concentration; Table 1) using Brilliant II SYBR® Green qPCR master mix as provided by the manufacturer (Stratagene). Conditions for qPCR (40 cycles) were as follows: 95°C for 30 s, 60°C for 1 min, then 72°C for 1 min. Each reaction was performed with three replicates and the average taken for each animal, with *n*-values representing the number of animals.

**Table 1:** **Primers for real-time reverse quantitative polymerase chain reaction**.

**Gene**	**Forward primer**	**Reverse primer**	**Size (bp)**	**Accession no**
Collagen I	TGGCTGCACGAGTCACACCGG	GGGAGGTCTTGGTGGTTTTG	68	NM_053304.1 (Keane et al., 2007; Mizuno et al., 2009)
Collagen III	GGTCAGCCAGGTCGAGACGGATC	TGGGGCACCAGGAGAACCATTTT	72	NM_032085.1
Fibronectin	AGAGTGAGCCCCTGATTGGGAGGA	TCACCCTGCAAACCAACGGTCG	70	L00191
eNOS	GGATCCAGTGGGGGAAACTG	TGGCTGAACGAAGATTGCCT	123	NM_021838
iNOS	TGGCCTCCCTCTGGAAAGA	GGTGGTCCATGATGGTCACAT	95	U03699 (Edwards et al., 2004)
SOD	CCACTGCAGGACCTCATTTT	CACCTTTGCCCAAGTCATCT	218	NM_017050 (Duong et al., 2008)
p47 (phox)	CCAGCTCCCAGGTGGTATGAT	TCTTCACCTGGCTGTCATTG	178	AY029167 (Masamune et al., 2008)

Primers were designed and/or verified using the NBCI/ Primer-BLAST database (http://www.ncbi.nlm.nih.gov/tools/primer-blast/index.cgi?LINK_LOC=BlastHome). Testing included assessment of secondary annealing, mismatching or partial annealing to sequences other than the intended target. Correct product size for each primer set was confirmed by visualization of amplicons on Safegreen stained agarose gels. Primers provided in 3′–5′ sequence. Amplicon size is provided in base pair (bp). eNOS, endothelial nitric oxide synthase, iNOS, inducible NOS, SOD, superoxide dismutase, p47 (phox), NADPH oxidase subunit p47 (phox).

Cycle threshold (Ct) values represent PCR cycle number at which fluorescence emission data exceeded a threshold limit, with a lower number representing a higher level of expression. ΔCt values were calculated by normalizing to tyrosine 3-monooxygenase/tryptophan 5-monooxygenase activation protein, zeta peptide (YWHAZ) as an endogenous control, predetermined for stability using the geNorm reference gene selection kit as per the manufacturer's instructions (Primer Design Ltd, Southampton, UK). ΔCt values were compared for significant differences between ages and strain as detailed below, which drove subsequent calculation of fold variation between the chosen reference (defined as a set value of 1) and other groups using the 2^−(Δ^ΔCt) method (Livak and Schmittgen, 2001), with range values determined using the formula 2^−(Δ^ΔCt + SEMΔCt). Calculation of range values was similarly applied to the chosen reference, providing range values around the set value of 1.

### Statistical analysis

Results are expressed as mean ± standard error of mean (SEM). Analysis was conducted using IBM Statistical Package for the Social Sciences (SPSS; v20, IL, USA) and Prism v 6.0a (GraphPad Software Inc. CA, USA). Preliminary analysis of data to identify strain and treatment effects was undertaken using a univariate general linear model (GLM) against the fixed factors of age and strain with gender entered as a covariate. A Brown-Forsythe test was used to determine if there were any differences in the variance, and if so, the data was log-transformed before statistical analysis. Unless otherwise stated, age and strain effects were not influenced by gender (*P* > 0.05). Results were evaluated by Two-Way ANOVA followed by Bonferroni *post-hoc* analysis, driven by the major effects and/or interactions.

## Results

### Body weight, cardiac hypertrophy, blood pressure, and renal function

Baseline animal phenotypic data is presented in Table 2. LPK animals weighed significantly less than Lewis at both ages, with females weighing less than males in both strains. Heart index, left ventricular index, kidney weight index and SBP were greater in the LPK than Lewis at both ages studied, but did not change with age within either the LPK or control strain. Plasma creatinine and urea were markedly elevated in the LPK compared with age-matched Lewis, and showed an age-dependent increase within the LPK strain.

**Table 2:** **Body and tissue weights, systolic blood pressure and renal function variables**.

**Parameter**	**12-week-old**		**18-week-old**	
	**Lewis**	**LPK**	**Lewis**	**LPK**
BW (g)	286.0 ± 41.6	185.0 ± 18.7^a^	330.0 ± 55.9^b^	199.2 ± 26.4^a*^
HI (%)	0.3 ± 0.01	0.5 ± 0.02^a^	0.3 ± 0.02	0.5 ± 0.02^a*^
LVI (%)	0.2 ± 0.01	0.4 ± 0.04^a^	0.2 ± 0.01	0.4 ± 0.02^a*^
KI (%)	0.9 ± 0.01	9.6 ± 0.43^a^	0.8 ± 0.01	9.1 ± 0.34^a*^
SBP (mmHg)	129.7 ± 2.0	204.6 ± 10.4^a^	119.2 ± 3.5	215.7 ± 3.5^a*^
P_Cr_ (μmol/L)	19.3 ± 1.8	42.5 ± 3.5^a^	21.2 ± 0.7	110.0 ± 6.2^a*^^,^ ^b*^
Urea (mmol/L)	8.1 ± 0.2	26.3 ± 2.0^a^	6.9 ± 0.3	39.7 ± 1.4^a*^^,^ ^b*^

Results are expressed as mean ± SEM.

a P < 0.05 between 12 weeks Lewis and LPK.

a* P < 0.05 between 18 weeks Lewis and LPK.

b P < 0.05 between 12 and 18 weeks Lewis.

b* P < 0.05 between 12 and 18 weeks LPK.

Data was obtained from animals used for aortic PCR gene expression analysis (n-values as detailed for cohort (ii). BW, body weight; HI, heart index; LVI, left ventricle index; KI, kidney index; SBP, systolic blood pressure; P_Cr_, plasma creatinine; LPK, Lewis polycystic kidney rat.

### Vascular aortic ring contractility

Time control experiments showed that the aortic rings maintained their vascular response over the course of the experiment [NA maximum contractile response (R_max_) values at the start and finish of the experiment were: 12 weeks 3.4 ± 0.7 vs. 2.8 ± 0.7 contraction (N/g), 18 weeks 2.4 ± 0.3 vs.2.2 ± 0.5 N/g, *P* > 0.05 for all].

**Figure 1:**
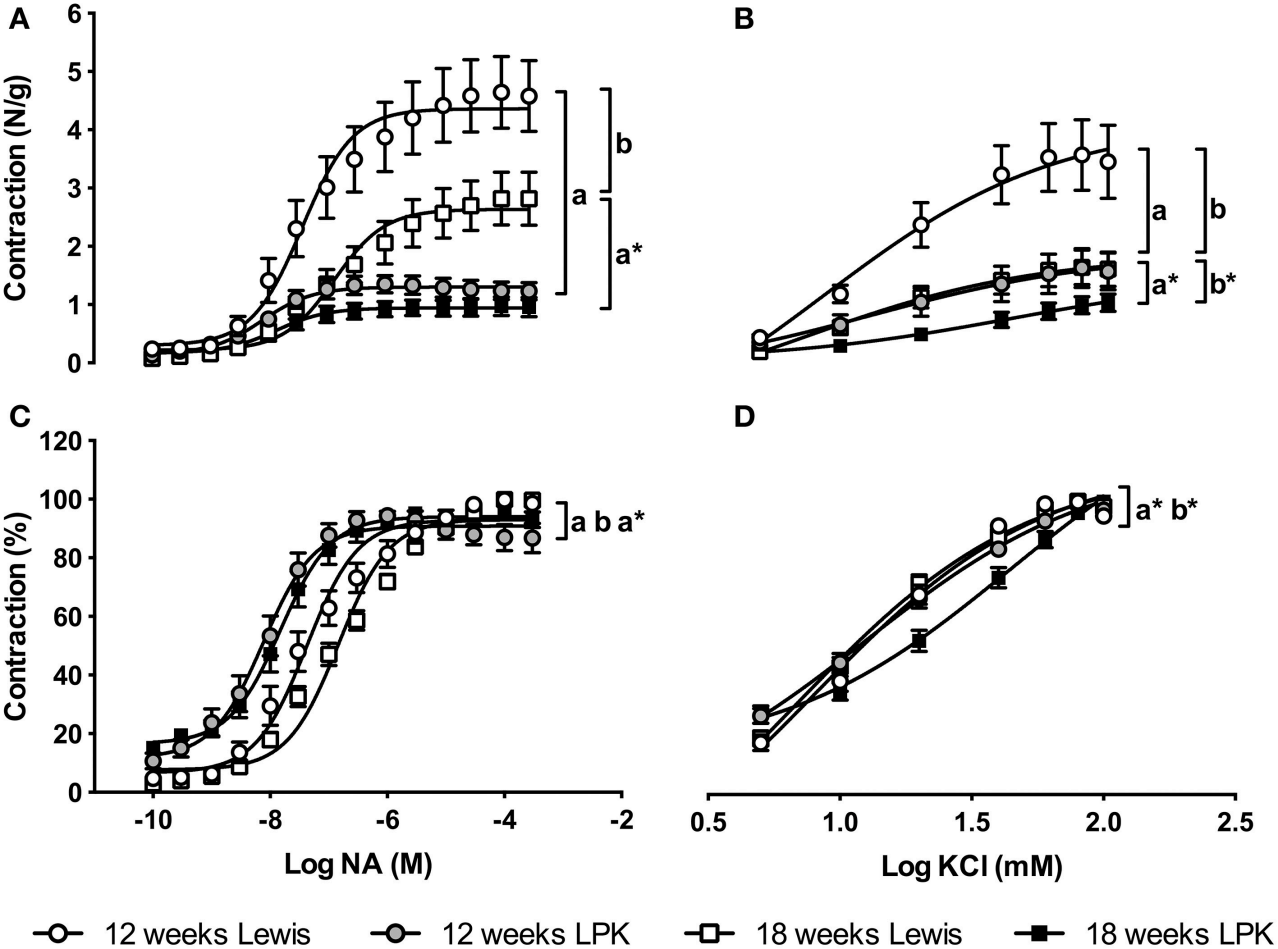
**Panels illustrate aortic contractile responses to cumulative additions of noradrenaline (NA; A,C) and potassium chloride (KCl; B,D) expressed as absolute force per aortic ring weight (N/g; A,B) and relative % of the vasoconstrictor (contraction %; C,D)**. Aortic contraction to NA was depressed in the Lewis polycystic kidney (LPK) rats at both ages, and showed an age related increase in sensitivity, while aortic contraction to KCl progressively declined with age. Significant differences between response curves are indicated *P* < 0.05 Lewis vs. LPK at (a) 12 weeks of age and (a^*^) 18 weeks of age. *P* < 0.05 for age effect in (b) Lewis and (b^*^) LPK.

Contraction concentration-response curves to NA were blunted in the LPK relative to aged-matched Lewis, with an age effect (reduction) evident in Lewis but not LPK rats (Figure 1A). This was reflected in a lower R_max_ for LPK at both 12 and 18 weeks vs. age matched controls, and an age effect for R_max_ in the Lewis (Table 3). AUC for NA was also significantly less in the LPK rats at 18 weeks vs. age matched controls (*P* = 0.01; Table 3). There was a leftward shift in the % contraction response relationship to NA in the LPK aorta at both age points compared to Lewis rats (Figure 1C), but this was seen as a significant difference in the EC_50_ in the 18-week-old age group (Table 3), suggestive of a progressive increase in sensitivity toward the α-adrenergic agonist.

**Table 3:** **Parameters describing the concentration-response curve to various pharmacological agents in 12 and 18 weeks of age Lewis and LPK aortic rings**.

	**EC_50_ (M) (%)**		**R_max_**		**AUC**	
	**Lewis**	**LPK**	**Lewis**	**LPK**	**Lewis**	**LPK**
**12-WEEK-OLD**						
NA	10.8 × 10^−8^ ± 59.1 × 10^−9^	13.3 × 10^−9^ ± 55.2 × 10^−10^	4.4 ± 0.6 N/g	1.3 ± 0.2 N/g^a^	17.7 ± 2.7 N/g	6.3 ± 0.7 N/g^a^
KCl	8.5 ± 1.6	13.4 ± 2.6	3.7 ± 0.6 N/g	1.9 ± 0.4 N/g^a^	3.1 ± 0.4 N/g	1.5 ± 0.3 N/g^a^
ACh	49.1 × 10^−9^ ± 14.4 × 10^−9^	83.7 × 10^−8^ ± 37.7 × 10^−8^	91 ± 3%	80 ± 8%	253 ± 19%	187 ± 27%
SNP	32.7 × 10^−10^ ± 62.6 × 10^−11^	19.9 × 10^−9^ ± 90.0 × 10^−10^^a^	99 ± 0.4%	92 ± 6%	286 ± 11%	244 ± 24%
ACh+L-Arg	96.3 × 10^−9^ ± 16.8 × 10^−9^	32.0 × 10^−9^ ± 10.2 × 10^−9^^c^	79 ± 6%	71 ± 4%	205 ± 22%	227 ± 17%
ACh+L-NAME	12.1 × 10^−7^ ± 35.1 × 10^−8^^d^	45.3 × 10^−10^ ± 25.1 × 10^−10^	27 ± 7%^d^	26 ± 3%^d^	75 ± 17%^d^	69 ± 9%^d^
ACh+L-Arg+L-NAME	28.9 × 10^−8^ ± 22.2 × 10^−8^^e^	25.4 × 10^−9^ ± 16.5 × 10^−9^	57 ± 6%^e^	40 ± 10%	210 ± 32%^e^	135 ± 38%
**18-WEEK-OLD**						
NA	19.2 × 10^−8^ ± 49.3 × 10^−9^	24.3 × 10^−9^ ± 78.1 × 10^−10^^a*^	2.6 ± 0.4 N/g^b^	1.0 ± 0.1 N/g^a*^	9.5 ± 1.6 N/g^b^	4.5 ± 0.7 N/g^a*^
KCl	7.6 ± 1.5	67.8 ± 25.0^a*^^,^^b*^	1.8 ± 0.3 N/g^b^	1.4 ± 0.2 N/g	1.5 ± 0.2 N/g^b^	0.9 ± 0.1 N/g
ACh	10.2 × 10^−7^ ± 38.2 × 10^−8^^b^	57.6 × 10^−9^ ± 24.3 × 10^−9^^a*^	96 ± 2%	57 ± 9%^a*^^,^^b*^	210 ± 20%	160 ± 20%
SNP	10.0 × 10^−9^ ± 81.7 × 10^−11^	68.6 × 10^−10^ ± 14.5 × 10^−10^	95 ± 3%	70 ± 9%^a*^^,^^b*^	232 ± 12%	174 ± 20%^b*^
ACh+L-Arg	21.1 × 10^−8^ ± 16.0 × 10^−8^^c^	52.8 × 10^−9^ ± 25.8 × 10^−9^	82 ± 6%	75 ± 3%	271 ± 29%	252 ± 17%^c^
ACh+L-NAME	26.8 × 10^−7^ ± 22.5 × 10^−7^	53.7 × 10^−11^ ± 16.6 × 10^−11^	50 ± 6%^b^^,^^d^	32 ± 5%^d^	113 ± 19%^d^	100 ± 24%
ACh+L-Arg+L-NAME	12.0 × 10^−8^ ± 59.2 × 10^−9^	82.6 × 10^−9^ ± 34.9 × 10^−9^	75 ± 4%^e^	40 ± 7%^a*^	238 ± 29%^e^	140 ± 28%

Results were evaluated by Two–Way ANOVA followed by Bonferroni post-hoc analysis. Results are expressed as mean ± SEM.

a P < 0.05 between 12 weeks Lewis and LPK.

a* P < 0.05 between 18 weeks Lewis and LPK.

b P < 0.05 between 12 and 18 weeks Lewis.

b* P < 0.05 between 12 and 18 weeks LPK.

c P < 0.05 treatment effect within strain and age (ACh vs. L-Arg).

d P < 0.05 treatment effect within strain and age (ACh vs. L-NAME).

e P < 0.05 treatment effect within strain and age (L-NAME vs. L-Arg + L-NAME).

NA, noradrenaline; ACh, acetylcholine; SNP, sodium nitroprusside; L-Arg, L-arginine; L-NAME, N_ω_-nitro-L-arginine methyl ester; EC_*50*_, effective concentration at 50%; R_max_, maximum response; AUC, area under the curve; LPK, Lewis polycystic kidney rat.

Maximal aortic smooth muscle contraction to the depolarizing signals of high K^+^ was significantly lower in LPK compared with Lewis and progressive age-dependent changes were evident in both strains (Figure 1B). LPK R_max_ and AUC values for KCl were lower than controls at 12 weeks but not 18 weeks. A progressive decline in these values was seen with age in Lewis rats only (Table 3). Contraction responses to KCl expressed as a % were rightward shifted in the 18-week-old LPK relative to age-matched Lewis and younger LPK (Figure 1D), and a higher EC_50_ value was also observed in these animals (Table 3).

### Vascular aortic ring relaxation

**Figure 2:**
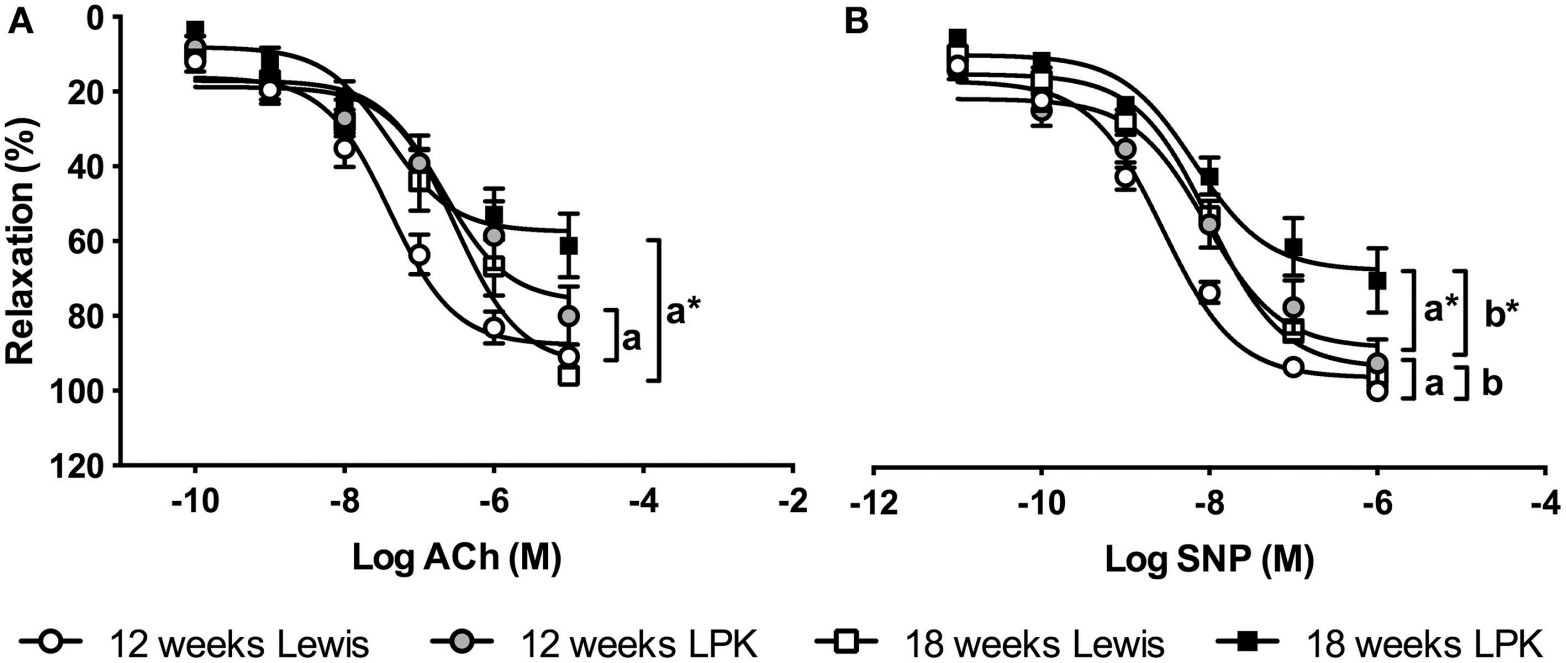
**Aortic relaxation responses in vessels precontracted with 1 μM phenylephrine (PE) to cumulative additions of (A) acetylcholine (ACh), (B) sodium nitroprusside (SNP), examining endothelium-dependent and -independent relaxations, respectively**. The Lewis polycystic kidney (LPK) rats show impaired endothelium-dependent and -independent relaxations at both ages. *P* < 0.05 Lewis vs. LPK at (a) 12 weeks of age and (a^*^) 18 weeks of age. *P* < 0.05 for age effect in (b) Lewis and (b^*^) LPK.

Aortic endothelium-dependent relaxation curves were blunted in the LPK compared to age-matched Lewis (Figure 2A). The 18-week-old LPK R_max_ values for ACh were significantly less than age-matched Lewis and 12-week-old LPK rats (Table 3). EC_50_ was lower in the 18-week-old LPK compared to controls, with the Lewis rats having an age-dependent decrease in endothelium-dependent relaxation in terms of EC_50_ values (Table 3).

Aortic endothelium-independent relaxation curves in response to SNP were rightward-shifted in the LPK and 18-week-old Lewis (Figure 2B). EC_50_ values in 12-week-old LPK rats were greater than age matched Lewis rats and R_max_ was significantly reduced in 18-week-old LPK vs. Lewis rats of the same age and 12-week-old LPK (Table 3). AUC for SNP also showed an age related decline in the LPK.

### Vascular aortic nitric oxide synthase functionality

**Figure 3:**
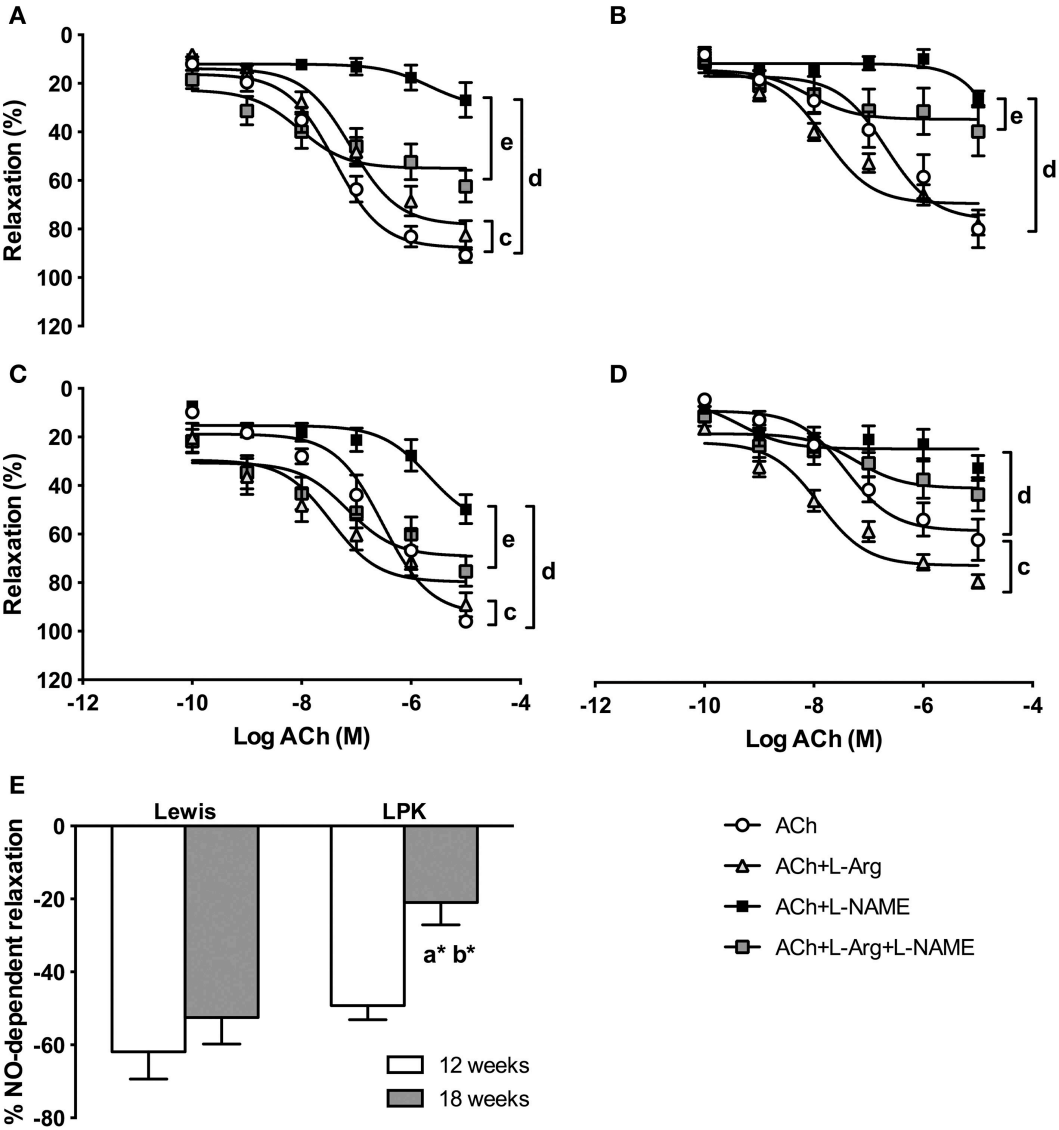
**Panels illustrate aortic relaxation responses in vessels precontracted with 1 μM phenylephrine (PE) to cumulative addition of acetylcholine (ACh) in (A) 12 week old Lewis, (B) 12-week-old Lewis polycystic kidney (LPK) rats, (C) 18-week-old Lewis, and (D) 18-week-old LPK**. Panels illustrate control (ACh only) and ACh responses in the presence of L-arginine (L-Arg, 10 mM), N_ω_-nitro-L-arginine methyl ester (L-NAME, 10 μM) or both. Percentage (%) relaxation was determined as % inhibition of PE contraction. **(E)** illustrates overall nitric oxide (NO)-dependent component of ACh-induced relaxation, calculated as the difference in R_max_ response to ACh before and after incubation with L-NAME. Results are expressed as mean ± SEM. (a^*^) *P* < 0.05 Lewis vs. LPK at 18 weeks of age, (b^*^) *P* < 0.05 for age effect in LPK. (c) *P* < 0.05 treatment effect within strain and age (ACh vs. L-Arg), (d) *P* < 0.05 treatment effect within strain and age (ACh vs. L-NAME), (e) *P* < 0.05 treatment effect within strain and age (L-NAME vs. L-Arg + L-NAME).

1. L-Arg: ACh-mediated relaxation was slightly blunted in the 12-week-old Lewis when aortic rings were preincubated with L-Arg (Figure 3A) but this was not accompanied by any significant differences in EC_50_, R_max_ or AUC (Table 3). By contrast, in the 18-week-old Lewis, L-Arg shifted the ACh concentration response curve to the left (Figure 3C) with a significantly lower EC_50_. In the LPK, addition of L-Arg to the organ bath at 12 weeks resulted in a smaller EC_50_ measure relative to responses with ACh alone (Table 3). At 18 weeks in the LPK animals, the addition of the NOS substrate did not alter the EC_50_ but did result in a greater AUC compared with responses in the presence of ACh alone (Figure 3D, Table 3).
2. L-NAME: L-NAME markedly blunted the ACh-mediated responses in all groups (Figures 3A–D), R_max_ was lower in the presence of L-NAME than in the presence of ACh alone at all strains and ages (Table 3). AUC was also significantly lower at all ages and strains other than LPK rats at 18 weeks (Table 3).
3. L-Arg and L-NAME: The combination of both NOS substrate and inhibitor in the organ chamber attenuated NOS inhibition and shifted the ACh concentration-response curve downward toward greater recovery of the vasorelaxation except in the 18-week-old LPK (Figures 3A–D). This resulted in a reduction in the ACh EC_50_ of the young Lewis and increase in the R_max_ and AUC values of both 12- and 18-week-old Lewis (Table 3), but this was not reflected in the results for the LPK of either age.

The NO-dependent component of ACh-mediated relaxation was significantly reduced in the aged LPK relative to both 18-week-old Lewis and younger LPK (Figure 3E).

Mean values for plasma creatinine and urea for this cohort of animals [cohort (i)] was as follows: creatinine (Lewis 12 weeks 27.1 ± 0.3, Lewis 18 weeks 27.4 ± 1.5, LPK 12 weeks 66.4 ± 2.7, LPK 18 weeks 123.0 ± 5.0 μmol/L) and urea (Lewis 12 weeks 6.2 ± 0.5, Lewis 18 weeks 7.7 ± 0.3, LPK 12 weeks 29.5 ± 1.0, LPK 18 weeks 43.6 ± 0.9 mmol/L) and both parameters showed the same age and strain effects as for the animal cohort presented in Table 2, being elevated in the LPK compared with age-matched Lewis, and an age-dependent increase for both urea and creatinine occurring within the LPK strain.

### Plasma nitric oxide and vascular aortic calcification

Plasma levels of NO^−^_2_, NO^−^_3_ and total NO did not significantly differ between Lewis and LPK and measures did not change with age (Table 4). Thoracic aortic calcium content however was markedly increased in the LPK vs. Lewis animals, and there was an age-dependent increase in aortic calcium observed in both strains (Table 4).

**Table 4:** **Plasma nitrite, nitrate, total NO levels and calcium aortic content in 12- and 18-week-old Lewis and LPK rats**.

**Parameter**	**12-week-old**		**18-week-old**	
	**Lewis**	**LPK**	**Lewis**	**LPK**
NO^−^_2_ (μM)	32.7 ± 6.4	27.3 ± 6.5	25.4 ± 4.5	22.9 ± 4.9
NO^−^_3_ (μM)	145.6 ± 25.7	206.3 ± 24.8	208.3 ± 15.8	230.8 ± 16.8
Total NO (μM)	178.3 ± 20.8	233.6 ± 21.1	233.6 ± 16.8	253.7 ± 16.8
Aortic Ca content (μmol/g)	24.3 ± 4.3	39.5 ± 4.9^a^	40.5 ± 1.9^b^	57.5 ± 3.4^a*^^,^^b*^

Results are expressed as mean ± SEM. Results were evaluated by Two–Way ANOVA followed by Bonferroni post-hoc analysis.

a P < 0.05 between 12 weeks Lewis and LPK.

a* P < 0.05 between 18 weeks Lewis and LPK.

b P < 0.05 between 12 and 18 weeks Lewis.

b* P < 0.05 between 12 and 18 weeks LPK.

NO^−^_*2*_, nitrite; NO^−^_*3*_, nitrate; NO, nitric oxide; Ca, calcium; LPK, Lewis polycystic kidney.

### Vascular aortic quantitative polymerase chain reaction mRNA expression

The ΔCt values for each age and strain for the respective genes are provided in Table 5. Preliminary statistical analysis of strain and treatment effects was used to determine which specific groups were compared for reporting as fold variation [2^−(Δ^ΔCt)] (Livak and Schmittgen, 2001). Chosen reference groups were (i) Lewis (both age groups combined) when assessing strain only effect; (ii) 12-week-old data (both strains combined) when assessing age only effect, (iii) 12-week-old Lewis (when assessing both a strain and age effect).

**Table 5:** **ΔCt values for aortic mRNA gene expression in 12- and 18-week-old Lewis and LPK rats**.

**mRNA gene expression**	**12-week-old**		**18-week-old**		**Two-Way ANOVA adjusted *P*-value**	
**(ΔCt)**	**Lewis**	**LPK**	**Lewis**	**LPK**	**Strain**	**Age**
Collagen I	−2.58 ± 0.66	−3.54 ± 0.13	−2.22 ± 0.22	−4.06 ± 0.78	0.009	0.854
Collagen III	−2.52 ± 0.49	−2.61 ± 0.11	−2.59 ± 0.04	−4.40 ± 0.73	0.029^§^	0.031
Fibronectin	0.08 ± 0.78	−0.13 ± 0.40	1.93 ± 0.26	−1.43 ± 0.35	0.005	0.606
eNOS	3.02 ± 0.37	2.97 ± 0.02	2.97 ± 0.30	2.13 ± 0.37	0.163	0.167
iNOS	5.91 ± 0.28	5.01 ± 0.40	5.08 ± 0.76	4.79 ± 0.20	0.223	0.286
SOD	−2.23 ± 0.20	−2.22 ± 0.27	−3.83 ± 0.26	−3.9 ± 0.11	0.899	< 0.001
p47 (phox)	4.26 ± 0.32	3.97 ± 0.19	4.53 ± 0.30	1.84 ± 0.28	< 0.001	0.006

Δ cycle threshold (ΔCt) values representing threshold PCR cycle number normalized to endogenous control gene YWHAZ, with a lower number representing a higher level of expression. Results are expressed as mean ± SEM. Results were evaluated by Two–Way ANOVA followed by Bonferroni post-hoc analysis.

§ Indicates significant gender effect. LPK, Lewis polycystic kidney; eNOS, endothelial nitric oxide synthase; iNOS, inducible NOS; p47 (phox), NADPH oxidase subunit p47 (phox); SOD, superoxide dismutase.

**Figure 4:**
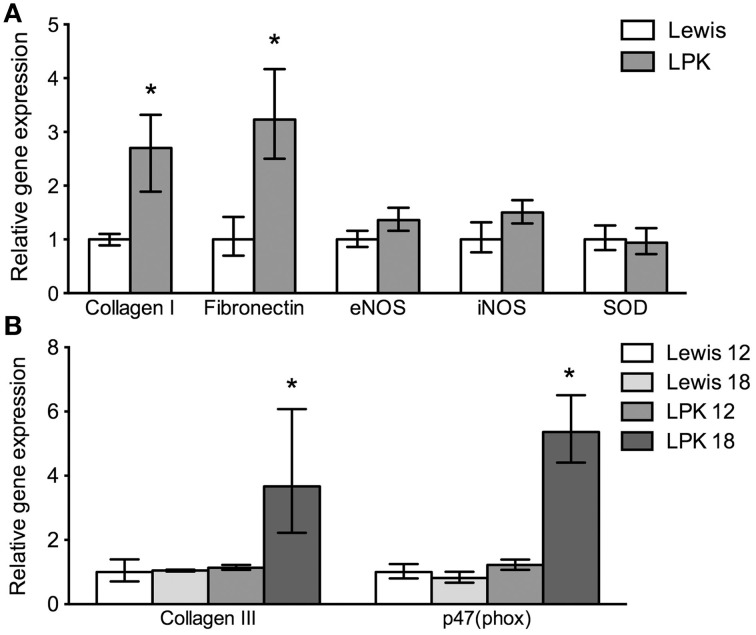
**Relative gene expression levels for aortic markers of fibrosis, NO synthesis and oxidative stress**. Gene expression was normalized to YWHAZ as an endogenous control and then ΔCt values compared for significant differences between ages and strain (Table 5). Figure presents fold variation and range values for selected results for **(A)** *strain effect*, with data expressed relative to Lewis (both age groups combined) and **(B)** *strain and age effect*, with data expressed relative to 12-week-old Lewis. ^*^Significant difference (*P* < 0.05) between **(A)** LPK and Lewis controls or **(B)** LPK 18-week-old animals and all other groups. LPK, Lewis polycystic kidney; eNOS, endothelial nitric oxide synthase; iNOS, inducible NOS; p47 (phox), NADPH oxidase subunit p47 (phox); SOD, superoxide dismutase.

Fold variation data is presented in Figure 4. Collagen I expression showed a significant strain effect, but no age effect, being greater overall in LPK aorta (Figure 4A). Collagen III expression showed a significant age and strain effect, being significantly increased in the 18-week-old LPK animals (Figure 4B). Collagen III was also influenced by gender, with 18-week-old male LPK animals having higher collagen levels than 18-week-old female LPK. Fibronectin levels were significantly influenced by strain, being greater in the LPK animals (Figure 4A). While there was no age effect, there was a strain x age interaction (*P* = 0.01) which was the result of 10-fold higher levels of fibronectin mRNA in 18-week-old LPK vs. 18-week-old Lewis. Inducible NOS (iNOS) and endothelial NOS (eNOS) levels were not significantly different between groups (Figure 4A). Superoxide dismutase (SOD) gene expression did not show a strain effect (Figure 4A) but there was an age effect, being increased in the 18-week-old animals overall (fold difference 12-week-old animals: 1.0 ± 0.1 vs. 18-week-old animals 3.1 ± 0.3). Expression levels of mRNA for the NADPH oxidase subunit p47 (phox) showed significant strain and age effects due to higher levels of p47 (phox) mRNA in the 18-week-old LPK animals (Figure 4B).

## Discussion

The incidence of cardiovascular disease is markedly increased in patients with advanced kidney disease and is associated with changes in vascular function (Sutliff et al., 2011). We have previously shown that the thoracic aorta from the LPK rodent model of CKD exhibits significant remodeling (Ng et al., 2011; Ameer et al., 2014) and in this study we aimed to determine if aortic vascular functional responses declined in parallel with measures of renal dysfunction and examined potential underlying mechanisms. We show that aortic vessel responses are altered in the LPK model of CKD, and that a temporal deterioration in function is present, manifesting as endothelium-dependent and -independent deficits that associate with markers of advancing CKD. We provide evidence that the LPK rat aorta exhibits a significant reduction in its ability to contract or relax in an *in-vitro* experimental paradigm, displays increased sensitivity to a sympathetic agonist, functional loss of NOS activity, a progressive increase in aortic calcification, increased expression of mRNA for the fibrosis markers collagen I, III and fibronectin, and increased mRNA levels of the NADPH p47 (phox) subunit, an indicator of increased oxidative stress.

Altered aortic responsiveness to vasoconstrictors and impairments of the vessel contractile capacity were apparent and progressive in nature in LPK rats. Studies investigating the effect of renal failure on aortic reactivity to vasoconstriction stimuli have proven disparate, with some studies showing reduced responsiveness and others showing enhanced responses (Rascher et al., 1982; Zimlichman et al., 1984; Meggs et al., 1986; Sutliff et al., 2011). In this study, we were able to show that a combination of both defects exist in the LPK, whereby adrenergic-mediated vasoconstriction is more sensitive in the LPK, as indicated by reduced EC_50_ measures; however maximal responses to NA and high K^+^ were not attainable, reflected in the blunted R_max_ and AUC in the LPK.

The hypersensitivity to sympathetic pressors that we observe in this study may be related to deficiency of vasodilators such as NO (Chang and Stevens, 1992), prostacyclin or kinins, vascular structural alterations secondary to pre-existing hypertension, or retention of uraemic toxins (Beretta-Piccoli et al., 1982). Intriguingly, heightened sensitivity to the sympathetic agonist in the LPK was not associated with similar increases in the sensitivity to muscle depolarization with high K^+^. This contrasts with reports in the adenine-induced renal failure model, where renal failure induced by adenine consumption combined with dietary modifications to markedly accelerate vascular calcification resulted in increased sensitivity to the depolarizing signals of high K^+^, yet unaltered sensitivity to α-adrenergic stimuli (Sutliff et al., 2011). This dissimilarity may be due to the acute time course over which severe kidney failure and vascular calcification was achieved in the adenine model, which as noted by the authors, prevented remodeling of the vessel wall that would otherwise occur in a more naturally progressive form of CKD (Sutliff et al., 2011).

In the LPK, maximal impairment of adrenergic vasoconstriction was attained early during the course of CKD, while high K^+^ induced vasoconstriction declined further with age. An age related decline in aortic compliance was also seen in the Lewis animals, and in both strains we saw progressive vascular calcification, with greater calcium levels in the LPK animals at both time points. This is consistent with previously described negative associations between arterial compliance and magnitude of vascular calcification (Niederhoffer et al., 1997; Sutliff et al., 2011) and substantiates aortic calcification as a feature of both arterial aging (Cecelja and Chowienczyk, 2012) and the underlying pathology of CKD (Sarnak et al., 2003). Changes in the structural components that make up the vessel wall are also believed to be an important mechanism underlying arterial stiffness (Zieman et al., 2005) and our demonstration of higher mRNA levels of collagen I, III and fibronectin in the current study support this premise, consistent with our previous findings of both vascular remodeling and increased pulse wave velocity in the LPK rodents (Ng et al., 2011). In this study, we also show that the LPK rats exhibited cardiac and left ventricular hypertrophy. As cardiac ventricles and the arterial tree constitute a coupled biological system (Munoz and Sacco, 1997), these cardiac remodeling changes could be linked to the demonstrated alteration in aortic compliance and its influence on blood pressure wave state, both of which increase cardiac work load, and consequently cause the heart to adapt by remodeling (Chen, 2012).

In addition to stable components that make up the vessel wall, arterial stiffness can also be substantially influenced by dynamic factors such as endothelial cell signaling (Zieman et al., 2005). Data from the present study indicate an inability of the LPK rodent aortic tissue to achieve a maximal relaxation response under *in-vitro* conditions, and there was evidence of endothelial cell dysfunction, with impaired aortic vasodilator responses to ACh, which progressed in severity as the renal function declined. Endothelium-independent vasodilation, as elicited by the action of SNP on vascular smooth muscle was also impaired, and similarly deteriorated with age. Impaired endothelium-dependent and independent relaxations have been reported in a number of animal models (Wang et al., 1999; Karavalakis et al., 2008; Sutliff et al., 2011) and humans (Hand et al., 1998; Morris et al., 2001) with renal disease. Importantly, our observation of a decline in endothelium-dependent and-independent relaxations in parallel with the deterioration of renal function supports the hypothesis that systemic uraemic toxins can cause vascular damage and inflammation, leading to altered vascular function in CKD (Himmelfarb et al., 2002). The reduced capacity of the LPK aorta to relax is moreover consistent with a loss of arterial compliance, likely impacted by aortic calcification and stiffness as discussed above.

Our work and that of other indicates that ACh-mediated relaxation of large arteries from normotensive rodent aorta is predominantly mediated by NO (Spradley et al., 2013; Tanaka et al., 2015). Accordingly, our protocol involved studying the response of the aortic rings to ACh following preincubation with the NOS precursor L-Arg and the NOS inhibitor L-NAME. Our results show that L-Arg was able to improve the sensitivity to ACh-mediated relaxation in the 12-week-old LPK, as indicated by a lower EC_50_ in response to NOS substrate, and at 18 weeks of age in the LPK, L-Arg increased the AUC of ACh-mediated relaxation, noting however, this effect was not replicated in the EC_50_ data. Our data does therefore not support defects in the uptake of the precursor L-Arg through the endothelial cationic transporter (Schiffrin et al., 2007) as being a moderating mechanism of endothelial dysfunction in the LPK model. Interestingly however, we also observed aging effect in the Lewis animals, with a decrease in ACh sensitivity that was improved after L-Arg treatment in the older animals. These results suggest potential age and renal effects over ACh sensitivity that will benefit from future evaluation with L-Arg dose response curves.

Data from the present study showed that L-NAME attenuated the ACh-mediated relaxation in both strains; however, this was not associated with marked reductions in the AUC of the aged LPK relative to their AUC response in the absence of L-NAME, consistent with a progressive loss in functional NOS activity and therefore impaired NOS-mediated relaxation. Combination of the NOS substrate with the inhibitor, which assessed the relative sensitivity of NOS to both agents, revealed that NOS was able to compensate for the loss of function induced by the inhibitor in the young LPK but not in the older LPK. This indicates that the older LPK, with more progressed renal dysfunction, are hypersensitive to the enzyme inhibitor and recovery of any existing dysfunction of NOS was unattainable when disease reaches an advanced stage. This view is supported by the finding that the NO-dependent component of ACh-mediated relaxation temporally declined in the LPK. There were no differences in aortic eNOS mRNA expression levels, however we did not measure protein levels and the possibility that eNOS could be present in an inactive form warrants future investigation (Peterson et al., 2013). Future *in-vitro* studies which directly measure aortic NOS bioactivity and levels of both eNOS and the phosphorylation of serine 1177 of eNOS, reflecting the activated form of the protein (Boo et al., 2002) will greatly inform our overall understanding of the mechanisms driving changes in NO bioavailability and NOS sensitivity in large vessels associated with kidney disease. It has also been proposed that lack of compliance may contribute to a decrease in NOS activity, feeding into a negative cycle whereby structural changes further contribute to arterial stiffness (Zieman et al., 2005).

Systemic NO levels were not altered in this study, with no difference between groups in the plasma measures of NO^−^_2_, NO^−^_3_ and total NO. This is consistent with findings in the Han:SPRD Cy polycystic kidney disease rat model (Wang et al., 1999), however additional factors, including the contribution of other vascular beds will also influence systemic NO levels.

Endothelial dysfunction can alternatively arise from altered downstream responses to NO. Our data indicated a reduced sensitivity to the NO donor SNP in the young LPK animals, and an age effect was also evident, with 18-week-old LPK demonstrating reduced maximal relaxations. These data suggest that impaired NO/cGMP signaling, independent of NO production or bioavailability *per se*, may be a contributing factor to the observed decline in vasorelaxation responses in the LPK animals. Whether this is due to changes in the activity of soluble guanylate cyclase and/or levels of cGMP is yet to be established, however such mechanisms have recently been proposed to contribute to reduced vascular relaxation in obesity (Neves et al., 2014).

Vasodilation can be induced by ACh via mechanisms other than NOS activity, such as the release of prostaglandin and endothelial-derived hyperpolarizing factors which are resistant to the blocking effect of L-NAME (Kato et al., 1990; Wang et al., 2008; Giles et al., 2012). These pathways were not examined in the current study and specific tests to ascertain how their contribution was altered in the diseased state will be required.

Another pathway that could predispose to endothelial dysfunction is oxidative stress (Schiffrin et al., 2007). Various factors can stimulate NADPH oxidase (Schiffrin et al., 2007), leading to the generation of superoxide anion and thus contributing to endothelial dysfunction and vascular remodeling during hypertension (Touyz and Schiffrin, 2004). This is consistent with our findings of increased expression of the NADPH subunit p47 (phox) in the aorta of 18-week-old LPK animals. Our data has also shown an increase in the antioxidant SOD with aging. This was irrespective of strain and could be interpreted as a counter-regulatory mechanism that promotes a reduction in oxidative stress in the face of the vascular aging process, as has been previously documented (Guo et al., 2001). Future work that obtains direct estimates of both NADPH oxidase protein levels and SOD activity (Ding et al., 2012), as well as measures of vascular superoxide production (Tanaka et al., 2015) will provide a more complete understanding of the disease process, as will studies that seek to reverse or alleviate components of the vascular dysfunction that we have identified.

Reduced compliance of large arterial vessels in CKD patients has significant clinical implications, and our observation of multiple vascular structural and functional abnormalities in the LPK rodent model highlight the complex interaction of uraemia with the vasculature. Increased aortic stiffness, altered sensitivity to vasoconstrictor agents, impaired endothelium-dependent and –independent relaxation responses, and impaired NOS-mediated relaxation are all factors that could ultimately contribute to the transmission of greater pulsatile pressures and increased risk of end organ damage (Briet et al., 2011) and as such should therefore be considered important therapeutic targets to reduce cardiovascular risk in CKD.

## Author contributions

OA—Design of research, performed experiments; analyzed data; interpreted results of experiments; prepared figures; drafted manuscript; approved final version of manuscript and accountability for accuracy and integrity of the work. RB—Performed mRNA experiments; analyzed data; reviewed and revised manuscript; approved final version of manuscript and accountability for accuracy and integrity of the work. MB—Interpreted results of experiments; edited and revised manuscript; approved final version of manuscript and accountability for accuracy and integrity of the work. AA—Conception of research; edited and revised manuscript; approved final version of manuscript and accountability for accuracy and integrity of the work. JP—Conception and design of research; analyzed data; interpreted results of experiments, edited and drafted manuscript, prepared figures, revised manuscript and accountability for accuracy and integrity of the work.

### Conflict of interest statement

The authors declare that the research was conducted in the absence of any commercial or financial relationships that could be construed as a potential conflict of interest.

## Abbreviations

CKD: Chronic kidney disease
NO: nitric oxide
LPK: Lewis polycystic kidney
SBP: systolic blood pressure
NA: noradrenaline
PE: phenylephrine
ACh: acetylcholine hydrochloride
SNP: sodium nitroprusside
L-Arg: L-arginine
L-NAME: N^ω^-nitro-L-arginine methyl ester hydrochloride
AUC: area under curve
SEM: standard error of mean
eNOS: endothelial nitric oxide synthase
iNOS: inducible nitric oxide synthase
SOD: superoxide dismutase.
